# Regimes of Expectations: An Active Inference Model of Social Conformity and Human Decision Making

**DOI:** 10.3389/fpsyg.2019.00679

**Published:** 2019-03-29

**Authors:** Axel Constant, Maxwell J. D. Ramstead, Samuel P. L. Veissière, Karl Friston

**Affiliations:** ^1^Charles Perkins Centre, The University of Sydney, Sydney, NSW, Australia; ^2^Wellcome Trust Centre for Human Neuroimaging, University College London, London, United Kingdom; ^3^Culture, Mind, and Brain Program, McGill University, Montreal, QC, Canada; ^4^Department of Philosophy, McGill University, Montreal, QC, Canada; ^5^Division of Social and Transcultural Psychiatry, McGill University, Montreal, QC, Canada; ^6^Department of Anthropology, McGill University, Montreal, QC, Canada

**Keywords:** active inference, Markov decision process, social conformity, decision-making, deonticity, niche construction theory

## Abstract

How do humans come to acquire shared expectations about how they ought to behave in distinct normalized social settings? This paper offers a normative framework to answer this question. We introduce the computational construct of ‘deontic value’ – based on active inference and Markov decision processes – to formalize conceptions of social conformity and human decision-making. Deontic value is an attribute of choices, behaviors, or action sequences that inherit directly from deontic cues in our econiche (e.g., red traffic lights); namely, cues that denote an obligatory social rule. Crucially, the prosocial aspect of deontic value rests upon a particular form of circular causality: deontic cues exist in the environment in virtue of the environment being modified by repeated actions, while action itself is contingent upon the deontic value of environmental cues. We argue that this construction of deontic cues enables the epistemic (i.e., information-seeking) and pragmatic (i.e., goal- seeking) values of any behavior to be ‘cached’ or ‘outsourced’ to the environment, where the environment effectively ‘learns’ about the behavior of its denizens. We describe the process whereby this particular aspect of value enables learning of habitual behavior over neurodevelopmental and transgenerational timescales.

## Introduction: Toward a Mechanistic View of Regimes of Expectations

The theory of regimes of expectations (ROEs, see section “Glossary”) integrates research in cognitive psychology, theoretical neuroscience, and evolutionary and cognitive anthropology, to provide a novel perspective on longstanding questions about fundamental human social behaviors, such as the uniquely human propensity to cooperate, share intentions, and to coordinate action, enabling group decisions based on shared goals (Ramstead et al., 2016; Veissière, 2016; Veissière and Stendel, 2018).

A ROE is a set of expectations about states of the world characteristic of a given cultural group. Individual agents acquire ROEs in ontogeny through the selective patterning of attention and salience (Roepstorff et al., 2010; Tomasello, 2014), by leveraging shared expectations (often automatically and implicitly) to guide goal-directed behavior. Such practices lead agents to forage for information that is culturally marked as salient, which in turn resolves uncertainty about the world and underwrites the learning of context-specific expectations (e.g., preferences) that constitute a ROE (Tomasello et al., 2005; Ramstead et al., 2016).

### ROEs and Social Conformity

In social psychology, the notion of social conformity was originally formulated as *deference to the socially approved norm* (Asch, 1955) and was viewed as one possible response to social influence (Asch, 1956). Cultural evolutionary models of social conformity, in turn, emphasize the fitness-enhancing function of social conformity (for a review see Morgan and Laland, 2012). On this view, social conformity depends on a series of evolved dispositions to forage for social information; i.e., information acquired through social influence (Kendal et al., 2018). Examples of such dispositions include the tendency to allocate attention to agents marked as socially relevant and the tendency to copy and imitate these agents (Boyd and Richerson, 1988; Henrich, 2015; for reviews see Laland, 2018), e.g., people who elicit epistemic trust (Fonagy and Allison, 2014). Social conformity is cast as a learning strategy effective in uncertain environments, one allowing individuals, especially naive newcomers, to zero in on the locally adaptive behavior. Cultural evolutionary theory thereby operates a shift in the study of social conformity, from the study of external influences on conformity (e.g., normative conformity), to the study of conformity as an *adaptive strategy* to cope with *uncertain environments*.

Regimes of expectations are the set of expectations to which we implicitly appeal when we ask ourselves the question ‘what should one do?’ in context; where ‘one’ can be viewed as a ‘generalized other,’ understood as the internalized attitudes and dispositions that are characteristic of a given community (Mead, 1934). ROEs may be useful to explain social conformity by producing automatic behavioral responses in high-order, rule-governed contexts (explaining, e.g., why some people stop or do not stop at a red traffic light at 4 AM with no one around). This is because ROEs prescribe typical, admitted forms of social behavior that include one’s own attitude, as a member of that community, along with preferences, values, goals, etc., that are characteristic of that community.

Preferences constitute, in part, the ‘cultural’ affordances (i.e., set of action possibilities that are relevant and available to an agent, Gibson, 1979; Rietveld and Kiverstein, 2014) that are learned implicitly through natural pedagogy (Csibra and Gergely, 2011), by inferring what the authoritative, trusted, prestige-laden others from one’s in-group would expect one to do in a relevant situation; e.g., ‘what would mother want me to do?’ (Henrich and Gil-White, 2001). Environmental cues indicating prestige can be described as possessing epistemic authority (see section “Glossary”), acquired via inferences about actual and generalized agents who might enforce their normative or deontic status. Receptivity to such environment cues rests on evolved biases for cultural learning, which allow humans to be unusually and effortlessly skilled at zooming in on salient, contextually relevant cues with high normative or deontic value.

### ROEs and Human Decisions Making

The concept of ROE is also useful to account for human decision-making (see section “Glossary”). Humans have a marked propensity to attend to each other’s actions to figure out ‘what would a typical other do,’ even when this is maladaptive or irrational (Cook et al., 2011; Belot et al., 2013; Naber et al., 2013). Humans are highly skilled at outsourcing their decision-making and reasoning to trusted, ‘prestigious’ others they have come to associate with epistemic authority. Listening to a charismatic, trusted religious figure known to have “healing abilities,” for example, has been shown in experimental settings to inhibit frontal executive brain function in religious Christians (Schjoedt et al., 2011).

The presence of authoritative others has been documented to guide automatic decision making through a process that cognitive anthropologists call credibility enhancing displays (Norenzayan et al., 2016). While it may present a challenge to verify empirically, recent ‘interactionist’ accounts of the social nature of solitary reasoning (Mercier and Sperber, 2017) strongly suggest that automatic and deliberate decision-making are underwritten by the (implicit and explicit) outsourcing of culturally-appropriate modes of action-readiness to actual and generalized others.

### Outline of the Argument

Expectations that make up a given regime of expectations (ROE) are realized or instantiated through properties that are both internal (i.e., encoded in the brain) and external (i.e., encoded in the environment) to the agent (Badcock, 2012; Constant et al., 2018a,b; Badcock et al., 2019; Ramstead et al., 2019a,b). For instance, the expectations that drive appraisal at a traffic light include individually learnt, brain-based expectations (e.g., knowing how to drive, having learned the traffic laws), as well as on material cues in the environment, such as traffic lights, the presence of other pedestrians, cars, etc. Hence, ROEs are “encoded in multiple levels and sites: in the hierarchical neural networks, in the organism’s phenotype [...], and in patterned sociocultural practices and designer environments” (Ramstead et al., 2016, p. 17). However, the theory of ROEs still lacks a proper formal (i.e., computational) basis. This renders the theory of ROEs difficult to implement in empirical studies of phenomena to which it naturally lands itself, such as social conformity and human decision making.

In this paper, we describe a formal model of ROEs that could be used to systematize and study human social conformity and decision-making. In §2 we base our model on the active inference scheme (see section “Glossary”) in theoretical neuroscience (Friston, 2005; Friston et al., 2017a), as applied to adaptive behavior and navigation in (discrete state space) environments (Kaplan and Friston, 2018)^1^. Active inference is akin to traditional reinforcement learning schemes that aim to optimize behavior as a function value, reward, or utility maximization (Rescorla and Wagner, 1972). However, in active inference, this optimization is done with respect, not to utility, but rather to some (Bayesian) beliefs, or expectations that the agent entertains about its environment. This process involves various internal computations and action-perception cycles, as well as environment modifying actions (Bruineberg et al., 2018; Constant et al., 2018b). Hence, active inference naturally lands itself as a suitable computational framework for describing ROEs.

In §3, the mathematical formalism of active inference is applied to the theory of ROEs to show that social conformity and human collaborative decision making naturally follow from the individual optimization of Bayesian expectations (e.g., learning, or model fitting) and from the enactment of those expectations via environment-modifying actions that leave durable and informative traces (e.g., deontic cues) in the shared econiche. This entails a circular causality between the internalization of ‘shared’ Bayesian expectations via social learning, individual policy selection and decision making, and the implicit production of cues that support social conformity and the internalization of shared expectations back again.

## An Active Inference Model of Regimes of Expectations

Active inference speaks to earlier Kantian and Cartesian ideas about how we navigate our worlds phenomenally, by encountering it as it appears to our senses and cognitive architecture. These ideas can be traced through psychology, via analysis by synthesis (Yuille and Kersten, 2006), perception as hypothesis testing (Gregory, 1980), epistemological automata (MacKay, 1956), and action-perception cycles (Dayan et al., 1995; Friston et al., 2013). Contemporary formulations (including the one presented here) emphasize the roots of active inference in American pragmatism and ecological psychology (e.g., Dewey and Gibson); frameworks in which perception is cast as a means for behavior; which, in turn, rests heavily on the leveraging of information directly perceivable in the environment (e.g., affordances) (Bruineberg and Rietveld, 2014; Ramstead et al., 2016; Constant et al., 2018b).

Adaptive agents like humans tend to alter their behavior when they find themselves in surprising situations. For example, as one stirs one’s cooking pasta, and water spills, one spontaneously moves one’s hand away to avoid (unwanted burning sensations). This corresponds to the avoidance of sensory states that provide sensory evidence against one’s expectations about the integrity of your body. While one’s reflexes turn out to fulfill one’s expectations about preferred and typical bodily states, and in so doing generate evidence for those same expectations, the pre-emptive withdrawal of one’s hand avoids surprises in the future. The action that was selected was determined by the sorts of sensations expected with or without an avoidance maneuvre.

**FIGURE 1:**
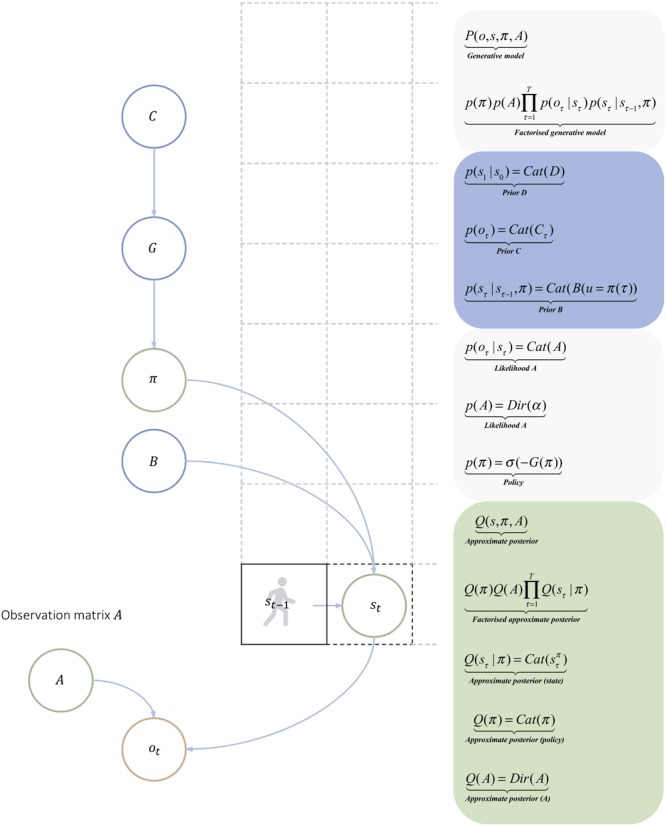
Generative model for discrete state-space navigation. The above graphical model shows the relations among the different quantities involved in action policy selection. We refer the reader to Friston et al. (2015) for a detailed discussion of these quantities, update rules, and for the variational Bayesian method used to update the approximate posterior. The generative model specifies the agent-environment relation in terms of a joint probability _P(o,s,π,A)_. Blue circles contain the quantities known by the agent, the green circles contain the quantities that must be inferred; namely, the action policy, the states of the world that generate observations, and future states upon which observations depend. The orange circle represents an observation. The generative model comprises a likelihood and prior over states, policies, and parameters. The observation matrix A specifies the likelihood of outcomes under each state of the world. Here, each location in an 8x8 world where the Markov decision process, takes place (shown for illustrative purposes, as gray dashed grid). The agent acts by changing its state (i.e., from _s_τ_-1 to s_τ__), which depends on the selected policy _π_. The most likely or valuable policies are those that minimize expected free energy G, which depends on C – a cost function that attributes a prior cost, when the agent encounters surprising states, say, encountering red locations. C implements pragmatic value discussed in the main text. D refers to the prior expectations about the starting location, and Cat and Dir refer to the form of the distribution (categorical or Dirichlet). The transition matrix, or prior beliefs about transitions, B encoded the possible transitions that can be engaged by the agent, given its allowable policies. A policy corresponds to a sequence of actions; for example, going up, down, left, right, stay. In the above figure, the agent is at _s_τ_-1_, and has to infer the policy, say, to reach _s_τ__.

According to active inference, expectations are continuously updated according to Bayesian principles that underlie perceptual synthesis; namely, attention and perception (Helmholtz, 1962; Dayan et al., 1995; Kersten et al., 2004; Knill and Pouget, 2004; Yuille and Kersten, 2006; Hohwy, 2016; Palacios et al., 2017). Active inference assumes that adaptive behavior can be described as realizing the predictions of a generative model (see section “Glossary”) of the agent’s ongoing sensorimotor engagement with its environment. Here, states of the environment define a generative process (see section “Glossary”), which refers to the dependencies among true states of the world and observations (Figure 1). The generative model is an internal probabilistic model of the causal factors that generate the agent’s sensory observations, now and in the future. The generative model is used by the agent to select actions that maximize the Bayesian model evidence (see section “Glossary”) expected under the agent’s generative model; or equivalently, to minimize the expected surprise or uncertainty about outcomes (Friston, 2013; for a discussion of biological plausibility, see Friston et al., 2010). The mathematical details of active inference may look complicated; however, they follow in a straightforward way from a generic model of any world that can be cast in terms of discrete states. For completeness, we will briefly describe the mathematical formalism behind active inference and the implicit belief updating, noting that these details do not change with the domain of application – and are not necessary to understand the basic ideas.

The relationship between the generative model and the generative process is that the agent learns or finesses its expectations about the states of the generative process based on the sensory observations it samples – as it navigates the world and selects policies (i.e., sequences of actions) as a function of its newly updated expectations. Crucially, these expectations are grounded by prior beliefs: if an agent expects to encounter green locations, then it will learn the parameters of the generative model accordingly, to approach green locations – and policy selection will be geared toward sampling evidence consistent with expectations about outcomes, in the fashion of a self-fulfilling prophecy. In short, it will look as if the agent prefers green locations if, and only if, the environment (generative process) can fulfill these expectations.

### Policy Selection

Reinforcement learning schemes and other similar utilitarian formulations face a glaring problem as an account of human choice behavior. Although uncertainty gets into the game via constructs like Bayesian decision theory (Daw et al., 2005; Colas et al., 2010; Solway and Botvinick, 2012), utilitarian formulations miss the point that the resolution of uncertainty is, in and of itself, valuable (Howard, 1966). In other words, there is an intrinsic motivation for everything we do that is completely independent of expected utility (Deci and Ryan, 1985; Barto et al., 2013). This intrinsic motivation underwrites epistemic foraging, and the fundamental drives for an embodied engagement with the world.

For example, most of our choices are driven by their epistemic affordance or ability to resolve uncertainty about states of affairs in the world; for instance, ‘what would happen if I did that?’ (Oudeyer and Kaplan, 2007; Schmidhuber, 2010). This resolution of uncertainty is a prerequisite to any behavior that can be adequately described in terms of maximizing expected utility. On this view, expected utility theory and its psychological concomitants (such as reinforcement learning) could be viewed as the legacy of behaviorism that predominated in the early 20th century.

In active inference, policy selection not only requires Bayesian belief updating; it also entails the imperatives for action. In brief, actions are considered more likely if they maximize the evidence expected under the consequences of that action. Mathematically, this means selecting actions that optimize expected free energy. This expected free energy comprises different terms, such that action policy selection depends on: (i) the potential for information gain about future states of the world (i.e., epistemic value or affordance), and (ii) to potential for fulfilling preferred sensory outcomes (i.e., pragmatic value or affordance).

Here, the generative model that is used to study adaptive behavior under active inference has a ‘Markovian’ form, in the sense that it explains navigation and observations as a function of state transitions in a Markov decision process (MDP) (cf. Figure 1). The environment in which the MDP unfolds is the generative process; i.e., how unobserved states of the world generate observations: e.g., one’s position in an 8x8 grid world and a visual feature that would be sampled at that location, say red or green. In this simple example, the generative process could generate (exteroceptive) outcomes that could be red or green and (proprioceptive) sensations that could signal some location information.

Active inference describes how these sensory observations are used to infer the true causes of sensations (i.e., location and color) – and how to actively sample this world to reduce uncertainty and realize prior preferences. Selecting optimal policies under Markovian models rests on evaluating expected variational free energy (see section “Glossary”) (Elfwing et al., 2016; Mirza et al., 2016). This quantity involves the expectations and preferences contained within the generative model. The expected variational free energy G of a policy _π_ at time _τ_ can be expressed as:

(1) $$\begin{array}{c} G\left(\pi ,\;\tau \right)=\underset{\mathrm{Epistemic}\;\mathrm{value}\;(\mathrm{state}\;\mathrm{and}\;\mathrm{paramters})}{\underset{︸}{E_{\tilde{Q}}\left[\ln Q\left(A\right)-\ln Q\left(A|s_{\tau },o_{\tau },\pi \right)\right]+E_{\tilde{Q}}\left[\ln Q\left(s_{\tau }|\pi \right)-\ln Q\left(s_{\tau }|o_{\tau },\pi \right)\right]}}-\underset{\mathrm{Pragmatic}\;\mathrm{value}}{\underset{︸}{E_{\tilde{Q}}\left[\ln p(o_{t})\right]}} \end{array}$$

Where:

$$\begin{array}{c} \tilde{Q}=Q(o_{{}_{\tau }},s_{\tau },A|\pi )=P(o_{{}_{\tau }}|s_{\tau })Q(s_{\tau }|\pi )Q(A) \end{array}$$

$\tilde{Q}$ is a counterfactual (‘what if’) posterior distribution over hidden states of the world s_τ_ at time τ expected by the agent, yet to be received observations o_τ_, and the parameters of the likelihood matrix A that contains expectations about outcomes given states of the world (e.g., greenness or redness of a particular location). In short, what is important for us is that under active inference (and discrete generative models), policies are selected as a function of expected free energy _G_, which comprises counterfactual expectations E_$\tilde{Q}$_ about what would happen if one were to enact a given policy π : i.e., ‘what observation o_τ_ I would make’ and ‘at what state s_τ_ I would be in.’ The epistemic value contains the expected information gain (i.e., uncertainty reduction) afforded a policy with respect to contingencies in the world (i.e., novelty), and the expected uncertainty reduction with respect to states of the world (i.e., salience) (Kaplan and Friston, 2018). Finally, the pragmatic value just is the value of a policy with respect to its potential of fulfilling preferred outcomes (i.e., potential for supplying expected sensory states).

This means that action is driven by expectations about ‘where I should be’ and ‘what I should perceive’ given some allowable actions. These expectations depend on the extent to which my observations will resolve uncertainty about the context ‘I find myself in’ and, at the same time, are consistent with the outcomes ‘someone like me’ would expect to encounter. In this setting, epistemic value is variously known as intrinsic motivation, Bayesian surprise, information gain or the value of information (Deci and Ryan, 1985; Schmidhuber, 2006, 2010; Oudeyer and Kaplan, 2007; Itti and Baldi, 2009; Barto et al., 2013). Conversely, pragmatic value is formally identical to utility or reward in economics and reinforcement learning (Ortega and Braun, 2011; Pearson et al., 2014; Bossaerts and Murawski, 2015).

Policy selection balances the pragmatic and epistemic affordance of action; namely, the extent to which the consequences of an action conform to the agent’s preferences (e.g., say, encountering green locations), and minimize uncertainty about the states of the world (e.g., its location). This amounts to the maximization of the pragmatic and epistemic values of action, which are associated with goal-seeking behavior and information seeking uncertainty-reducing behavior, respectively. The latter can also be construed as the epistemic affordance that underwrites curiosity and novelty seeking (Schmidhuber, 2010; Friston et al., 2015; Friston et al., 2017b; Kaplan and Friston, 2018).

### A DEEP Active Inference Model for ROEs

While the epistemic and pragmatic values depend on expectations about ‘what I should perceive’ and ‘where I should be’ under all plausible actions, the deontic value (or deontic affordance) of action relates to *shared* expectations; namely, expectations about ‘what one should do’ in a given situation – in other words, the most likely action that a typical agent (like me) would perform in a given setting. This allows for the tuning of one’s behavior to the expectations of others (usually, others like me) (Baker et al., 2011; Palmer et al., 2015), which may be broadly associated with the sharing of intentions (Tomasello et al., 2005; Tomasello and Carpenter, 2007).

By definition, the deontic value of an action relates to expectations that lie in the counterfactual depth of a typical other’s generative model: e.g., when one is alone at 4 AM at the red traffic light and engages in perspective taking to figure out whether one should cross the street or not. Interestingly, simulations of neural hermeneutics – namely, inferring an intended meaning – suggest that expectations encoded by generative models converge when engaging active inference in communicative setting; e.g., two artificial birds singing to each other by using the same generative model of birdsong (i.e., narrative) (Friston and Frith, 2015).

While neural hermeneutics with simple generative models of artificial birds may be fairly straightforward, in humans, inferring another’s intentions and selecting adequate policies – as a function of interpersonal and recursively nested expectations – must be much more computationally costly (e.g., take more time and energy). Yet, more often than not, we manage to automatically select consensual policies that lead to those actions that are expected by typical agents, even in time-pressured contexts. How is it that we manage to tune ourselves to such a complex nesting of expectations about an ‘ideal’ other, even when no other agent is around?

By relying on the environment to learn pro-socially optimal policies, agents save on computational cost (i.e., complexity) as the environment carries expectations about oneself and others’ expectations (Constant et al., 2018a,b). For instance, by constraining the action possibilities of pedestrians, drivers, and cyclists, the red traffic light indicates the behavior that both me and my fellow denizens are most likely to select. If I now incorporate this experience-dependent prior into my generative model, I have a way of automatically selecting policies that people ‘like me’ pursue; thereby minimizing my uncertainty (i.e., expected free energy). The idea here is that it is sufficient to use predictions about ‘how I would behave’ to explain ‘how you are behaving’; thereby finessing the infinite recursion of meta-metallization. Deontic cues (e.g., traffic lights), thus, effectively allows us to share a narrative, or ROE; even when alone.

**FIGURE 2:**
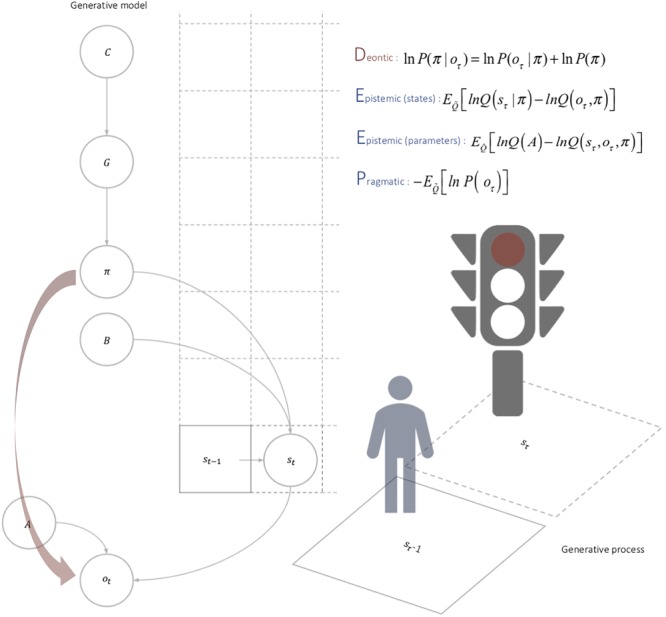
Deontic cues. In contrast to the epistemic and pragmatic value, the deontic value is specified directly by the observations currently at hand. In other words, it enables the agent to infer the best course of action quickly and efficiently based upon the information afforded by deontic cues. In sum, the deontic value of a policy depends on deontic cues, which are generated by the environment, and once learnt, constrain policy selection. However, the propensity of the environment to generate deontic cues itself depends upon the agent’s behavior. This circular causality brings something important to the table; namely, the ‘caching’ of beliefs about action in the environment; in particular, actions that were originally selected after observation were made and policies selected, for their epistemic and pragmatic value. Furthermore, because this ‘cache’ is shared by all agents that navigate the econiche, it enables a vicarious communication among agents, as the environment ‘learns’ about the creatures that shape it.

On this view, deontic value just is the value of action that can be inferred directly from deontic cues. Mathematically, deontic value corresponds to a direct likelihood mapping between policies and observations that can be inferred automatically in the presence of deontic cues, which function as a ‘cache’ encoded by the environment emerging from agents repeated action. In other words, expectations about ‘where I should go’ (epistemic value), ‘what I should perceive’ (pragmatic value), and ‘what one should do’ (deontic value) together constitute the architecture of the ROE learnt in ontogeny (see Figure 2). ROEs, then, can be viewed as guiding automatic social responses as a function of the Deontic, Epistemic (state), Epistemic (parameters), and Pragmatic (DEEP) values of an action policy. The depth of the DEEP model refers to the hierarchical depth of the generative model; either in terms of model states of the world or time points in the future (Table 1).

**Table 1:** DEEP model of ROEs.

	Axiology	Description
Deontic (external)	The (shared) value of policy endowed by a direct policy-outcome mapping, indicating ‘what one should do if?’	Deontic value: ln P(π|o_τ_) = ln P(o_τ_|π) + ln P(π) Deontic cue: P(o_i_| s_τ_) = $\frac{{\text{α}}_{i}}{\sum_{k}{\text{α}}_{k}}\;$ The deontic value is the direct likelihood of a policy given a particular outcome or cue. It corresponds to a likelihood term in the posterior beliefs about policies expressed in terms of likelihood and empirical prior, where the empirical prior just is the expected free energy (_-ln P(π)=G_). Referring to the generative model in Figure 1, one could imagine an arrow that goes directly from policies to a subset of outcomes, which reliably induce the policy (i.e., that bypass the optimization of beliefs about states, hence the direct mapping). In turn, the deontic cue is the probability of an outcome at any given state, which depends upon concentration parameters α that the environment learns as a function of how agents act on the world, which changes the value of the concentration parameters.
Epistemic (parameters) Epistemic (states) (internal)	The salience or information gain under a given policy, with respect to ‘where I should be if.’	E_$\tilde{Q}$_[lnQ(A) - lnA(o_τ_,s_τ_,π)] E_$\tilde{Q}$_[lnQ(s_τ_|π) - lnQ(o_τ_,π)] The expected uncertainty reduction with respect to the states that would generate observations. It captures the information gain expected by observing an outcome (this is also the mutual information between expected outcomes and their causes). Epistemic value can be decomposed into two terms: (i) the information gain for parameters (i.e., novelty), and (ii) the information gain for states (i.e., salience). This corresponds to the ‘information seeking’ dimension of behavior.
Pragmatic (internal)	The expected value of an outcome with respect to ‘what I should perceive if.’	-E_$\tilde{Q}$_[lnP(o_τ_)] : $\tilde{Q}$ = Q(o_τ_,s_τ_,A|π) The cost of engaging a given policy with respect to preferred outcomes. It drives the ‘goal seeking’ aspect of behavior.

### Modeling the Emergence of Deontic Cues Under Active Inference

The ability of environmental cues to solicit, for instance, stopping behavior at a red traffic light, rests on their epistemic value or affordance and a particular kind of context invariance. Intuitively, if a red traffic light could be turned to green whenever one wanted to cross a road, it would have a fairly weak, if any, influence on behavior (i.e., convey little deontic value). This suggests that the epistemic authority of an environmental cue (either artifactual, like a red traffic light, or human, like a traffic officer), rests on the propensity of that cue to retain its epistemic affordance when sampled.

Human agents pattern their environments, and in so doing produce human and non-human cues that modulate an agent’s foraging for socially relevant information (cf. cumulative downstream epistemic engineering Sterelny, 2003; Tomasello, 2014). The relevance of deontic cues rests on the fact that they consolidate (acquire potential for uncertainty reduction) as a function of the collective activity of agents of a given community (e.g., shared patterned cultural practice). Accordingly, we propose that deontic cues in the human niche emerge through the following circular causal process:

(i) Generic cues consolidate into deontic cues as a function of agential action on the social environment, which entails the structuring of inputs generated by the environment into a community-specific local social world (Clark, 2006).
(ii) Deontic cues come to indicate socially salient action, and to guide the agent’s active sampling of its world. This in turn patterns attention toward responding to deontic cues (i.e., informationally salient aspects of the niche), thereby leading to further active sampling consolidating deontic cues.

### Generic Cues Consolidate Into Deontic Cues Through Agential Actions on the Environment

As for the likelihood matrix of the generative model introduced in Figure 1, the generative process can be parameterized with a Dirichlet distribution. Each location contains a value for each possible sensory state (e.g., green and red), whose ‘redness’ and ‘greenness’ can be made more or less resistant to change (in the sense mentioned above). The robustness, or ability to resist changing as a function of agents’ actions can be modeled by increasing the concentration parameters of the Dirichlet distribution (as with the likelihood matrix of the generative model).

Effectively, this means that the environment will have to experience many instances of a particular combination of states and outcomes before the Dirichlet distribution changes. Technically, concentration parameters can be regarded as the number of events encountered and therefore determines the precision or robustness of the distribution in question. For simplicity, we will refer to concentration parameters as counts. On this view, the relative likelihood of an outcome is just the associated count, normalized by the sum of counts over all outcomes and the precision corresponds to the sum of counts.

This property of the deontic value means that one can treat it as an environmentally induced prior over policies that, unlike epistemic and pragmatic value, is *invariant to contextual uncertainty or prior preferences*. The environmental cue eliciting deontic value has a special property that it resists change when sampled. Hence, the environment offers these cues robustly or with precision. Because the count of the expectations (i.e., prior) in the generative matrix determines the relative probability of outcomes at each location, as the agent encounters a location, the environment will effectively learn the actions selected, on average, by agents. As a result, navigation will tend to move the expectations of the environment toward agents’ preferences.

Accordingly, when acted upon by multiple agents, deontic cues will move the expectations of the environment toward shared preferences; i.e., the preferences of the local community. Indeed, since the propensity of the generative matrix to change (i.e., its robustness) can also be modeled as the sum of concentration parameters at a location (i.e., prior precision), the more agents act on the environment, the more robust the cue will become; i.e., the higher the sum total of counts will be. The higher the total count, the less the environmental expectation changes as a function of an encounter with the agent, and consequently, the more likely the agent is to learn the value of the location and thereby sensitize its behavior to the deontic cues (i.e., update its action selection accordingly)^2^.

In this construction, deontic cues are high-fidelity cues from which agents learn, and according to which they structure their behavior. In cultural contexts, this suggests that the deontic structure of the environment (which comprises other agents, like the traffic officer, as well as non-human ‘agents’ like traffic lights) contextualizes action selection (Ramstead et al., 2016; van Dijk and Rietveld, 2016). Deontic cues thereby guide action toward the disambiguation of future states of the world; e.g., when you are at the intersection, and wonder whether the stationary car will remain stationary, you rely on the traffic light (i.e., its deontic value) to select your action accordingly. This means that an environmental cue will be deontic only in so far as agents ‘act out’ preferences (e.g., the red traffic light affording stopping, and the agent’s preference for stopping on the red). Put another way, deontic cues acquire the ability to solicit *pragmatic and epistemic habits* (Friston et al., 2016).

### Deontic Cues Flag Salient Action and Pattern Attention

The ROEs structures appraisals and automatic behavior upstream, via constraints on possible actions flagged by deontic cues in the generative process, and downstream, via policy selection within the generative model. Upstream constraints exist at the highest spatiotemporal scales of the architecture of expectations, in the sense that they are spatially extended (e.g., material setting vs. brain-based architectures), and change slowly, as they require the physical action of multiple agents (e.g., niche construction outcomes that emerge over hours and years vs. changes in neural connectivity that can change over milliseconds and seconds). This means that the higher levels of a ROE are more robust, as their physical implementation retains the traces of agents’ action over longer time scales. This allows for deontic cues encoded in the environment to be passed over generation via ecological and informational inheritance (Odling-Smee and Laland, 2011), and thereby allow the reproduction of attention, or epistemic foraging styles over ontogeny by shaping observations and states of the world encoded in an agent’s generative model.

From the point of view of active inference, one can distinguish two aspects of attention: namely, salience and precision (Parr and Friston, 2017). In the setting of policy selection, salience refers to the epistemic value or affordance of an action; i.e., the uncertainty reduction afforded by performing an action that may not have immediate pragmatic value, such as flipping a light switch to illuminate a room. When visual salience is high, a saccade will be elicited, for instance. In other words, our visual foraging or palpation is attracted to locations in the visual scene that afford the greatest information gain or resolution of uncertainty. In this setting, this salience is often referred to as Bayesian surprise. The other aspect of attention relates to learning (i.e., perceptual inference). Learning is modulated by the confidence put on sensory information (i.e., precision), which scores the extent to which a given sensory input is weighted as reliable or trusted; and is thought to be implemented by neuronal gain (for a comprehensive review, see Hohwy, 2013).

Although both components of attention are related, under active inference, they are clearly distinct. For instance, the selection of a location for a visual saccade (relating to salience and uncertainty reduction) is clearly distinct from the ‘downstream’ processing of the information made available by that saccade (relating to learning). One could consider the sort of attention associated with a more precise influence of deontic cues on policy selection as a more precise influence of sensory cues on belief updating.

The notion of ROE integrates those functions attributed to attention in relation to social cognition; namely, by providing a mathematical framework for modeling epistemic foraging styles, or what have been called ‘regimes of attention’ (Ramstead et al., 2016; Veissière, 2016). The notion of regime of attention is a cousin notion to the notion of ROE. Regimes of attention refer to a shared style of allocating attentional resources that characterizes a given cultural group; it marks certain information channels as especially reliable, leading to increase in neuronal gating allocated to that channel, and others not.

In the present setting, this attentional aspect reflects the mapping between deontic cues and value learned through action. Regimes of attention enable one to learn the epistemic value afforded by deontic cues, thereby relating epistemic value to attention. As the agent learns the epistemic value of a deontic cue, the deontic cue will elicit the action (e.g., an eye saccade) expected to most disambiguate the situation (e.g., saccade toward the gesture of the traffic officer), which will in turn elicit adaptive action (e.g., stopping), thereby consolidating the deontic cue (e.g., the officer gaining confidence in the ability of its gesture to elicit stopping).

## The Role of Regimes of Expectations in Social Conformity and Human Decision Making

The model on offer in this paper describes candidate mechanisms of automatized behavior that obtains via the learning of culturally specified deontic constraints. In our model, those mechanisms are the optimization of epistemic and pragmatic value of action and the consolidation of deontic cues in the environment. These cues pattern attention and, in return, steer the learning of the epistemic value afforded by deontic cues, and consequently action selection. In the remainder of this paper, we explore the manner in which our model resonates with phenomena related to social conformity and decision making in humans.

Crucially, we are not claiming that the aforementioned mechanisms necessarily results in decision making. Rather, based on key findings in the literature, we sketch a view on how these mechanisms may contribute to an explanation of certain features of social conformity and human decision making.

### Social Conformity From the Point of View of ROEs

In the introduction, we saw that social psychologists have defined the construct of social conformity as deference to the socially approved norms as one possible response to social influence; whereas cultural evolutionists have cast it as an adaptive learning strategy that allows individuals to zero in on locally adaptive behavior. These two perspectives on social conformity may be viewed as emphasizing two equally important dimensions of social conformity; respectively, the manner in which external social influence generates conformity, and the manner in which social conformity provides an adaptive advantage in certain environments.

Following Morgan and Laland (2012), views in social psychology and cultural evolution seem to intersect over the notion of *information foraging* as a drive for social conformity. The Asch experiment (Asch, 1955) is a standard experiment to study social conformity, which shows that under certain conditions, individuals can willingly defer to the group norm even in perceptual decision-making tasks (e.g., comparing and discriminating the size of visual targets). In Deutsch and Gerard’s (Deutsch and Gerard, 1955) variant of the Asch experiment, in which the streams of social influence were manipulated, the tendency to defer to group norm (normative influence) alone did not account for social conformity. There was an additional component that related to information foraging. As Deutsch and Gerard put it:

even if [one is] not normatively influenced, [one] may be influenced by the others in the sense that the judgments of others are taken to be a more or less trustworthy source of information about the objective reality with which he and the others are confronted. It is not surprising that the judgments of others […] should be taken as evidence to be weighed in coming to one’s own judgment. From birth on, we learn that the perceptions and judgments of others are frequently reliable sources of evidence about reality (Deutsch and Gerard, 1955).

The results of Deutsch and Gerard suggest that conformity is driven both by a utility maximizing component with respect to other people’s approval (the *normative* component), and a component based on the disposition to zero in on the correct solution by seeking information (the *informational* component). The informational component amounts to disambiguating one’s alternative interpretations by leveraging others’ interpretations, when they are deemed *reliable*. The balancing out of the utility maximizing (pragmatic) and information seeking (epistemic) value in action selection is precisely what drives an active inference agent’s behavior. In the variant of the Asch experiment, the deontic value is not made explicit, and might instead result from the ‘innate’ tendency to align one’s beliefs to that of trusted others, which come to signal highly reliable sources of information; i.e., come to function as deontic cues.

In effect, others and the deontic cues they produce are, more often than not, reliable sources of information that human learn to leverage over developmental and evolutionary timescales. While humans innately possess attentional biases to detect cues signaling health, vulnerability and status (Henrich, 2015; for a review, see Tomasello, 2014), many (though not all) cues will acquire stable deontic status within a particular group over development (e.g., learning to recognize specific symbols on a police uniform, or forms of dress denoting lower and higher socio-economic status).

The cultural evolutionary perspective on social conformity focuses on the study of conformity, operationalized as a learning rule that heavily relies on the acquisition of social information. In our model, one candidate mechanism whereby social learning obtains is via the attribution and leveraging of peoples and things’ deontic value. Deontic cues are a type of social information (i.e., information about community specific preferences outsourced to states of the niche).

Cultural evolutionary approaches seek to disentangle the effects of social learning (conveyed by demonstrators) and asocial learning (performed by individuals alone) to study the adaptive value of conformist responses. For example, a study by Morgan et al. (2012) found that the use of social information enhances performance in an object comparison task, which suggests that the use of social information is adaptive (cf. Javarone et al., 2016). The same study found that subjects are more likely to make a decision that is consistent with that of the majority, only when the number of demonstrators reaches a certain number – and when participants are uncertain of their own abilities (conveyed by the probability that subjects change their decision). This demonstration of the role of uncertainty in conformist responses resonates with the observation that the adoption of the majority’s behavior only obtains when the learner is naïve (Boyd and Richerson, 1988).

These findings are consistent with our model, which views conformity as the outcome of a dynamic process between information seeking and preference tuning responses. Scenarios wherein learners are naïve or uncertain – and wherein the number of replicators is high – can be framed as scenarios where the learners possess low confidence over prior beliefs concerning the state of affair in the world, and wherein the deontic value increases as a function of the increase in the precision of the environment (i.e., increase in the number of counts relative to the number of demonstrators performing the task).

Stable environments are by definition uninformative, in the sense that they are fully learnt under the agent’s generative model. Therefore, epistemic (i.e., informational) foraging will not be the main driver of behavior in such settings. Rather, the agent will act on the basis of her preferences (i.e., acting pragmatically), and will not learn from environmental cues. The process of conformity kicks in when the agent initially possesses imprecise beliefs about their environment, and therefore seeks to disambiguate her prior expectations about what sort of place she is operating in. In foraging for information, the agent will encounter deontic cues, the epistemic value of which she can learn, and which nuance her action selection.

### Comparison to a Closely Related Computational Model

An operational definition of social conformity based on the theory of ROEs under active inference may be stated as follows: social conformity is the state of affairs wherein the deontic value reifies the epistemic and pragmatic value of action relative to the preferences of a given cultural group embodied in deontic cues. Social conformity, therefore, may be viewed as a developmental process that obtains via *deontic constraints over the learning of expectations* that structure available action possibilities.

The view we offer in this paper is close in spirit to Toelch and Dolan (2015) formulation of informational and normative influences on social conformity in perceptual and value-based decision-making. Toelch and Dolan consider the process of perceptual decision making under social influence, casting perceptual decision making as the process of estimating environmental parameters through hierarchical Bayes (Mathys et al., 2011). In this setting, agents refine their estimations about the precision (i.e., inverse volatility) of environmental fluctuations. In Toelch and Dolan’s formulation, norms then guide behavior, in the sense that they come to influence the learning of precision parameters, thereby biasing perceptual decision making. In this setting ‘precision’ corresponds to ‘counts’ above, while ‘patterning of attention’ corresponds to what Toelch and Dolan call the ‘modulating of learning rate.’

Our model is consistent with Toelch and Dolan’s commitment to Bayesian inference and (precision or volatility) learning as an explanation for the influence of social norms. We go a step further by proposing that the emergence of deontic cues could account for some forms of norm formation, and that norms can be modeled in terms of ‘precision,’ though, on the side of the environment constructed by ‘people like us.’

Deontic cues emphasize the interplay between the formation of perceptual estimates of environmental parameters as described by Toelch and Dolan, the selection of actions based on the value of these estimates (among other things) that forms the basis of the epistemic and pragmatic value of action, and crucially, the looping or feedback influence of action on environmental parameters. Put another way, our model emphasizes the function of ‘salience’ (as opposed to precision, or inverse variance) as an attribute of prior beliefs (i.e., the estimates) influenced by deontic cues. What we call deontic cues, then, may be viewed as *external vehicles for norms*. Hence, the consolidation of deontic cues provides a rationale to model the manner in which norms consolidate and contribute to social conformity.

As things stand, our model remains purely theoretical. Careful experiments and simulation work will be needed to establish whether social influences in experimental settings, such as the Asch experiment, indeed stem from the effect of deontic cues.

### ROEs and Human Decision Making?

It is well-known in the empirical study of reasoning and decision-making in humans that models emphasizing the optimization of individual utility and competition between individuals – the so-called Machiavellian intelligence hypothesis (Barkow et al., 1995; Pinker, 1999; Dunbar, 2009) – do not explain the full range of observed reasoning and decision-making in humans. While human decision-making conforms to some canons of rationality [e.g., they mostly conform to the law of demand, and as the cost of fairness increases, people tend to do it less (Anderson and Putterman, 2006)]; other empirical investigations suggest that human cognition is optimized for *cooperation* through the sharing information with, and outsourcing of decision-making and policy selection to, other relevant human agents and cues in the environment (Henrich, 2015; Mercier and Sperber, 2017; for a review, see Tomasello, 2014).

The Ultimatum Game is a prime example of the propensity of human to outsource decision-making to third parties (Ensminger and Henrich, 2014). The game is about a monetary prize to be split amongst two players. The task of the first participant is to propose a proportion to share the winnings – say, splitting them 50–50; the task of the second player consists in accepting or rejecting this offer; this player’s role is crucial, for if they accept, the winnings are divided along the first player’s suggestion; otherwise, no winnings are distributed. From the point of view of classical economic theory, Nash equilibrium is for the first player to make a small offer (which maximizes individual utility), and for the second to accept anything offered – since something is better than nothing.

The comparisons of cognitive performance are striking in economic games between species. Unlike chimpanzees, human beings operate under the rubric of a ‘fairness psychology’ – a feature which makes them remarkably suboptimal at economic games that use the maximization of individual utility as a metric for success. Chimpanzees perform at Nash equilibrium; but humans, strikingly, do not; e.g., human players have a marked propensity to reject offers as the split departs from equality (Jensen et al., 2007; Prize et al., 2013). This makes little sense under the assumption that human beings are individual maximizers; but makes perfect sense if human beings instead tend to rely on third parties, to which they’ve learned to ascribe salience to support cooperation, and punish uncooperative behavior. On our model, this follows from the fact that the human social conformity becomes cooperative via the outsourcing of epistemic value, and is modulated by deontic cues (e.g., other agents).

In the 1960s it was found that human beings tend to reason by finding justifications for what they already believe – a phenomenon dubbed confirmation bias (Wason, 1960; Nickerson, 1998), but which is more accurately described as myside bias (Mercier and Sperber, 2017). In Wason’s famous card selection task, subjects are presented with four cards, each side of which has a number and a latter. The task consists in identifying which cards one must flip over in order to verify whether a given rule obtains (for example, ‘If there is an E on one side of the card, then there is a 2 on the other side of the card’). Human subjects do not seem to apply rules of inference; they seem to indicate the cards that are mentioned in the prompt. Only 10–20% of subjects respond correctly. Participants are much more likely to succeed in ‘isomorphic thematic’ versions of the task, that is, versions that map thematic rules over the logical rule.

Interestingly, human logical reasoning is optimized when (i) subjects are allowed to reason in groups – with success in performance rising from ∼20%, up to around 80% (Mercier and Sperber, 2017); and (ii), when the reasoning task is contextualized with less abstract or purely logical rules (e.g., If red, then stop, instead of If E then 2) – known as the content effect (Wason and Shapiro, 1971). Wason and Shapiro (Wason and Shapiro, 1971) hypothesized that increased relevance, operationalized by using terms relating to individuals’ experience, eases symbolic manipulation and reasoning. The optimization of cognitive performance and reasoning via the reliance on deontic cues (e.g., other humans and artifacts or representations that contextualize the task) can explain this observation. By outsourcing epistemic value to contextual deontic cues (context or others), one saves on computational cost of policy selection (Constant et al., 2018b), which eases decision making by allowing the agent to base its decision on robust regularities that pertain to one’s learned expectations relative to one’s social setting.

## Conclusion

We have proposed a normative model of ROEs (cf. Table 1.), which are typically used to explain human social conformity and decision making. We have attempted an explanation of how such architectures take hold in ontogeny, and how they are reproduced over generations. Finally, we have argued that social conformity could be defined as a state of affairs in which the value of action – evaluated under deep internal models – is cached or outsourced to an external, deontic level, and that human decision-making could be viewed as highly dependent on the reliance on those deontic cues and values.

Our model of ROEs may be of interest for more empirically-minded domains of research that study human culture and cognition. For instance, in health sciences, cultural psychiatry attempts to frame mental health in cultural terms; namely, by studying the influence of representations, symbols, artifacts, and culturally patterned practices on pathological behavior and psychiatric practice (for a comprehensive review, see Bhugra and Bhui, 2018). Our model may provide the basis for developing behavioral and cognitive markers of social integration on the background of which mental health diagnoses and treatments are performed.

Future research could address important, outstanding problems that relate to our model, such as how to model the specificity of expectations embodied in different environmental factors; e.g., how to study the distinction between the red traffic light, a parent, a religious artifact, etc. One putative consequence of our model, which could be pursued in future research, is the idea that the deontic value of action does not depend merely on isolated external cues, but often on an *ecology of cues*, which together form complex sets of expectations; e.g., the pattern of green, yellow, and red traffic lights, together with urban design of the intersection, pedestrian and cars, traffic laws and police, etc. Accordingly, the notion of regime of expectations may provide new computational schemes and strategies for fields such as cognitive ecology (Hutchins, 1995, 2010, 2014) and embodied and extended cognitive science (Clark and Chalmers, 1998; Chemero, 2009; Menary, 2010; Sutton, 2010; Kirchhoff, 2012); it is also relevant to areas of research like interactive design (Kirsh, 2013). Those fields study cognition as distributed over internal (e.g., brain) and external (e.g., environmental) processes, by offering an understanding of how action, perception, attention, and social learning in humans are structured in and around the epistemic niche (Clark, 2006).

A key question to further explore is how epistemic styles afforded by specific niches are inherited from one generation to the next, and how they can be finessed over ontogeny. Future research could relate our model to phenomena such as cumulative cultural evolution (Henrich, 2015) and cultural inheritance (Odling-Smee and Laland, 2011); especially in terms of epistemic power and epistemic authority such as vehicled by deontic cues. Future projects of ours include simulations of multiple interacting agents with different preferences and different generative process, to examine how patterned behavior emerges through collective actions on the environment. This will allow our model to be tested against behavioral data acquired, e.g., in navigation task in virtual environment.

## Author Contributions

AC, MR, SV, and KF contributed to the redaction of the manuscript. AC developed the framework and wrote the first draft. MR and SV contributed to the redaction in section titled. The role of regimes of expectations in social conformity and human Decision making KF supervised and wrote part of the formalism. All authors contributed to manuscript revision, read and approved the submitted version.

## Conflict of Interest Statement

The authors declare that the research was conducted in the absence of any commercial or financial relationships that could be construed as a potential conflict of interest.

## Footnotes

1 The terminology and formalism used in this paper refers to the Markovian interpretation of active inference. We refer both conceptually and technically minded readers who are unfamiliar with the relation between Markov decision processes and active inference to Friston et al. (2012) comprehensive introduction.

2 To model changes in the generative process as a function of the agent’s activity, one can implement an update rule for the locations. For instance, the update rule could be that for each encounter, the count of a preferred outcomes increases while the count of an undesired one remains unchanged, or even decreases, e.g., increasing the count of the green state and keeping that of the red state the same (for details see, and the MATLAB code and demo routines detailing the generative model of spatial navigation, which are available in the niche construction demo DEM Toolbox of the SPM open source software: http://www.fil.ion.ucl.ac.uk/spm/).

## Glossary

**Table:**

Concept	Definition
Regime of expectations	Set of expectations that structure action with respect to deontic, epistemic, and pragmatic values.
Deontic cue	Cues that induce behavior that remains unchanged over time, through reiterated action in the environment, while in turn being induced and maintained by the agent’s repeated actions in the environment.
Social conformity under active inference	The state of affairs wherein the deontic value comes to dominate policy selection and preferences in action and decision-making in a given cultural group. It obtains via the deontic constraints over the learning of expectations that, in turn, structure the available possibilities for action (i.e., epistemic and pragmatic value, or affordance).
Human decision-making	Unique propensity to rely on cooperative biases and to outsource policy selection to third parties (e.g., material or human cues).
Active inference	Process whereby agents garner Bayesian model evidence for their prior expectations about the world they inhabit. Technically, active inference entails the selection of action sequences that minimize expected free energy; i.e., minimize expected surprise (uncertainty) or, equivalently, maximize expected model evidence.
Prior expectations	Probability distribution over hidden states prior to observations.
Generative model	A joint probability distribution over environmental states and subsequent observations – usually specified in terms of prior expectations (about states) and the likelihood of observations given states. The generative model is a probabilistic model of the generative process.
Generative process	The actual process generating (observable) outcomes from (unobservable) states of the world.
Bayesian model evidence	Probability of observations given the generative model (i.e., independent of environmental states), known as the integrated or marginal likelihood (because one integrates or marginalizes over states of the model). The negative logarithm of model evidence is also known as surprisal.
Expected free energy	Functional of counterfactual expectations about states that comprise the epistemic, pragmatic, and deontic value of a policy. It quantifies the propensity of a policy to minimize expected surprise (a.k.a. surprisal or self-information) – or maximize expected Bayesian model evidence – in the future.
